# Association between Adiposity and Bone Mineral Density in Adults: Insights from a National Survey Analysis

**DOI:** 10.3390/nu15153492

**Published:** 2023-08-07

**Authors:** Yang Jiao, Juan Sun, Yuanmeng Li, Junduo Zhao, Jianxiong Shen

**Affiliations:** 1 Department of Orthopedics, Peking Union Medical College Hospital, Chinese Academy of Medical Science, Peking Union Medical College, Beijing 100730, China; sdujiaoyang@163.com (Y.J.); zjdzhaojunduo@163.com (J.Z.); 2 Department of General Surgery, Peking Union Medical College Hospital, Chinese Academy of Medical Science, Peking Union Medical College, Beijing 100730, China; pumc_sunjuan21@student.pumc.edu.cn; 3 Department of Endocrinology, Peking Union Medical College Hospital, Chinese Academy of Medical Science, Peking Union Medical College, Beijing 100730, China; lym2013sdu@126.com

**Keywords:** bone mineral density, android percent fat, visceral adipose tissue percent, gynoid percent fat, total percent fat, NHANES

## Abstract

Adiposity and bone mineral density (BMD) are closely associated. The aim of this research was to investigate the association between BMD and adiposity measures in adults, including gynoid percent fat (GPF), android percent fat (APF), total percent fat (TPF), visceral adipose tissue percent (VAT%), and total lean mass percent (TLM%). Participants (*n* = 11,615) aged 18 years and older were analyzed using data from the National Health and Nutrition Examination Survey (NHANES) spanning from 1999 to 2018. Associations between BMD and adiposity measures were investigated, and potential differences based on gender and age were explored. Significant negative associations were observed among TPF, APF, GPF, VAT%, and BMD in the fully adjusted models, while TLM% and BMD were positively associated. Stratifying by age and sex, TPF, GPF, and VAT% consistently demonstrated a negative correlation with BMD. In the young adult group, a TPF of 38.2% eliminated the negative correlation between BMD and TPF. Male BMD exhibited an inverted U-shaped relationship with APF, peaking at 35.6%, while a similar pattern was observed for the middle-aged group BMD and APF, with a peak at 31.7%. This large-sample research found a significant negative association between adiposity measures and BMD, providing valuable revelations regarding the intricate connection between adiposity and bone health.

## 1. Introduction

Obesity, a significant global public health concern, has reached alarming proportions in recent decades [1]. It is a chronic metabolic ailment characterized by excessive fat consumption and metabolic alterations [2]. The data presented by the World Health Organization (WHO) demonstrate that obesity not only poses substantial health risks as a chronic disease but also has a vital impact on various chronic non-communicable diseases and psychosocial disorders, such as type 2 diabetes, cardio-cerebrovascular conditions, respiratory ailments, and depression [2,3]. The accurate measurement and assessment of adiposity are imperative to comprehend its impact on health. While body mass index (BMI) has traditionally been the most widely utilized metric owing to its simplicity and convenience, recent scientific attention has shifted towards considering the role of fat distribution as a more precise and informative measure of adiposity and its associated health risks [4]. Consequently, dual-energy X-ray absorptiometry (DXA) emerges as a valuable tool for precise body composition analysis, including adiposity, lean mass, and bone mineral density (BMD) measurements [5,6].

Obesity, a multifaceted condition with far-reaching effects on physiological systems and organs, raises questions regarding its impact on bone health [7]. BMD, a crucial measure of bone “quantity”, exerts a significant influence on the progression of skeletal disorders like osteoporosis and osteopenia, which significantly heighten the risk of fractures and related morbidities [8]. Previously, it was widely believed that individuals with lower BMI were at a heightened risk of developing osteoporosis and fractures [9]. Multiple studies have also reported higher BMD values in individuals with obesity or higher BMI, seemingly supporting this perspective [1,7,8]. This finding can be attributed to the mechanical loading caused by increased body weight, which stimulates bone remodeling processes and ultimately leads to improved bone density as an adaptive response [10]. Nevertheless, the mechanical impact is not solely attributed to adipose tissue; it also extends to lean tissue. Many studies have demonstrated that lean tissue exhibits a more pronounced mechanical influence when it comes to enhancing BMD [11,12]. Furthermore, estrogen, which is present at higher levels in individuals with adiposity, has been proposed as a possible mechanism explaining the observed association between BMI and BMD [13]. Estrogen is indispensable to bone metabolism by promoting bone growth and suppressing bone resorption [14]. Additionally, the relationship between BMI and BMD appeared to exhibit sex-specific variations [15]. While some studies have found BMI and BMD were positively correlated in males, others have proposed that the safeguarding influence of adiposity on bone may not extend to the female population [16]. However, alternative perspectives have emerged from other studies indicating that adiposity can have detrimental effects on BMD in both males and females, with more pronounced consequences in males [15,17]. Age is another crucial factor that influences both BMI and BMD. With age, there is a gradual increase in the prevalence of adiposity accompanied by a decline in BMD [18,19]. Despite the growing recognition of the association between adiposity, lean tissue, and BMD, there is a dearth of large-scale studies examining this relationship, particularly concerning age and sex subgroup analyses.

Hence, it is crucial to explore the correlation between BMD and adiposity using large and diverse samples. To address this research gap, our study sought to utilize DXA as a precise measurement tool to comprehensively explore the association between regional adiposity, lean tissue, and BMD. According to the results of the National Health and Nutrition Examination Survey (NHANES) spanning from 1999 to 2018, we investigated a substantial and representative sample of the American population. Furthermore, subgroup analyses based on sex and age were conducted to comprehensively depict the various trends in these relationships.

## 2. Materials and Methods

### 2.1. Research Population

The 1999–2018 NHANES included 101,316 participants, and it aimed to evaluate the nutritional and health status of the American population. Among these participants, 59,204 individuals aged 18 and above were selected for inclusion in the present study. After excluding 28,591 participants with missing BMD data, 49 with missing total percent fat (TPF) data, 11,382 with missing android percent fat (APF) data, 7 with missing gynoid percent fat (GPF) data, and 7560 with missing visceral adipose tissue percent (VAT%) data, the final analysis included 11,615 participants aged 18–60 years with full data (as DXA scans were only performed on individuals less than 60 years old). The NHANES study obtained ethical clearance from the Research Ethics Review Board of the National Center for Health Statistics (Protocol #98-12, #2005-06, #2011-17). Further information can be found at https://www.cdc.gov/nchs/nhanes/irba98.htm (accessed on 23 May 2023).

### 2.2. Variables

Measurements of the adiposity and lean tissue variables, which included APF, GPF, TPF, VAT%, and total lean mass percent (TLM%), were conducted using Hologic Discovery Model A densitometers (Hologic, Inc., Bedford, MA, USA) equipped with Apex 3.2 software. Qualified radiologic technologists performed the measurements. The scanning analysis employed the Hologic APEX software, which defined two specific regions: android and gynoid (A/G). The android region encompassed by two lines was identified as the lower trunk region. The lower line was determined using the horizontal line of the pelvis, whereas the upper boundary was determined spontaneously using the software. The upper boundary of the gynoid region was positioned at a height 1.5 times greater than that of the android region below the horizontal line of the pelvis, and the lower boundary was established at twice the height of the android region. These lines were automatically positioned using the Hologic software. TPF represented the percentage of total fat mass in relation to the combined mass of total fat and lean tissue. VAT% is the ratio of the mass of visceral adipose tissue to the total mass of abdominal fat. The approximate interspace between the L4 and L5 vertebrae served as the point of measurement for assessing the VAT area, mass, and volume within the abdominal cavity. TLM% indicated the percentage of total lean mass (excluding bone mineral content) relative to the total DXA-measured mass.

The outcome variable, BMD, was measured by NHANES DXA using the Hologic Discovery Model A densitometers with Apex 3.2. BMD (g/cm^2^) was defined as bone mineral content (g) divided by the bone area (cm^2^) of the entire body. The specific information related to the measurement of body composition and BMD using DXA can be found on the website https://wwwn.cdc.gov/nchs/nhanes/Search/DataPage.aspx?Component=Examination (accessed on 23 May 2023), specifically within the section titled “Dual-Energy X-ray Absorptiometry—Whole Body”.

Our analysis incorporated a diverse range of additional variables obtained from the NHANES, including race, age, sex, income-to-poverty ratio, BMI, arm circumference, waist circumference, hypertension, hyperlipidemia, diabetes, vigorous work activity, and smoking status. Within the NHANES laboratory segment, we included a diverse array of metabolic indicators, including total cholesterol (TC), triglycerides (TG), low-density lipoprotein (LDL), high-density lipoprotein (HDL), blood urea nitrogen, creatinine, and serum uric acid. For detailed information protocols, visit the website of NHANES at https://wwwn.cdc.gov/nchs/nhanes/continuousnhanes/default.aspx (accessed on 23 May 2023), within the section titled “Demographics Data, Examination Data and Questionnaire Data”.

### 2.3. Data Analysis

The mean ± SD (standard deviation) was employed to report continuous variables, while percentages were used to represent categorical variables. Statistical analyses were conducted using the R package and Empower Stats. The relationships between BMD and TPF, APF, GPF, VAT%, and TLM% were evaluated using multivariate regression models. Statistical significance was defined as *p*-values below 0.05. The models were adjusted for age, race, sex, hypertension, hypercholesterolemia, diabetes, vigorous work activity, and smoking status. Stratified subgroup analyses were conducted to examine specific subpopulations, with sex and age as variables. The age variable was further categorized into two groups: “middle-aged adult” (40–60 years) and “young adult” (18–40 years).

## 3. Results

This study included 5768 female and 5847 male participants. Detailed baseline characteristics of all participants, divided by gender, are presented in Table 1. The BMI values for males (28.36 ± 5.80 kg/m^2^) and females (28.68 ± 7.28 kg/m^2^) were similar. However, in relation to fat distribution, females exhibited higher levels of TPF, APF, and GPF than males. Conversely, the VAT% and TLM% were lower in females than in males. Regarding metabolic indicators, males demonstrated higher levels of TG, LDL, creatinine, blood urea nitrogen, and serum uric acid than females. Conversely, the TC and HDL levels were lower in males than in females. The incidence of hypertension, hyperlipidemia, and diabetes was higher among male participants than among their female counterparts. Moreover, the incidences of smoking and vigorous work activity were found to be higher in males compared to females.

In the fully adjusted model, significant correlations were found between various body composition indexes (TPF, APF, GPF, VAT%, and TLM%) and BMD. Specifically, there were negative associations between TPF, APF, GPF, and VAT% with BMD, while TLM% showed a positive association with BMD (TPF: β = −0.001, 95% CI: −0.003–−0.001; APF: β = −0.000, 95% CI: −0.001–−0.000; GPF: β = −0.002, 95% CI: −0.002–−0.001; VAT%: β = −0.002, 95% CI: −0.002–−0.002; and TLM%: β = 0.001, 95% CI: 0.000–0.001). The subsequent sex-stratified analysis demonstrated the persistence of significant correlations between the body composition indexes and BMD in males. However, in females, both the APF and TLM% did not exhibit significant associations with BMD. Further stratification by age revealed that all associations remained significant in the middle-aged adult group. Conversely, in the young adult group, no significant associations were observed between APF and TLM% with BMD. These findings are summarized in Table 2.

**Figure 1:**
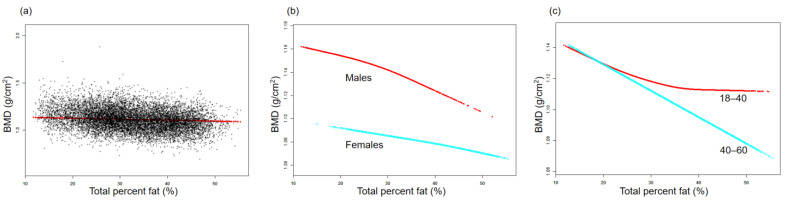
Relationship between BMD and total percent fat. (**a**) Each black dot on the graph denotes a single sample and the red line represents the fitted line for all participants. (**b**,**c**) Association of BMD with TPF stratified by sex and age. Baseline characteristics were adjusted. The subgroup analyses were performed without adjusting for sex and age.

**Figure 2:**
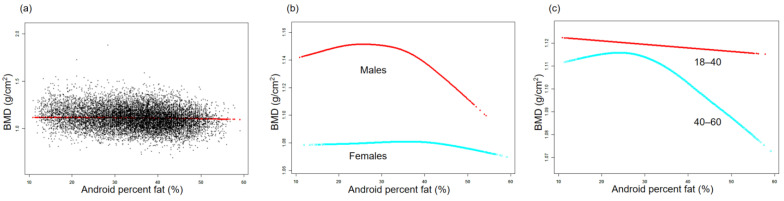
Relationship between BMD and android percent fat. (**a**) Each black dot on the graph denotes a single sample and the red line represents the fitted line for all participants. (**b**,**c**) Association between BMD and APF stratified by sex and age. Baseline characteristics were adjusted. The subgroup analyses were performed without adjusting for sex and age.

**Figure 3:**
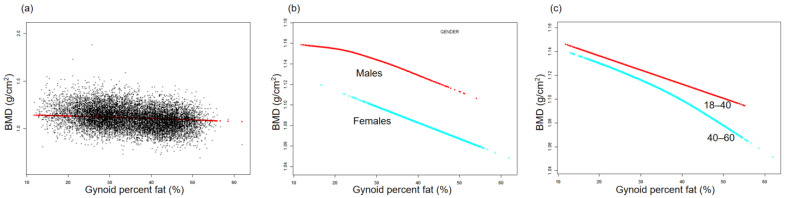
Relationship between BMD and gynoid percent fat. (**a**) Each black dot on the graph denotes a single sample and the red line represents the fitted line for all participants. (**b**,**c**) Association between BMD and GPF stratified by sex and age. Baseline characteristics were adjusted. The subgroup analyses were performed without adjusting for sex and age.

**Figure 4:**
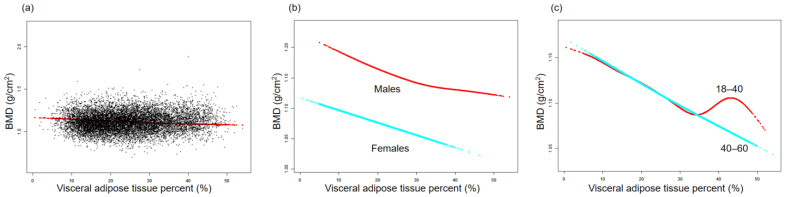
Relationship between BMD and visceral adipose tissue percent (%). (**a**) Each black dot on the graph denotes a single sample and the red line represents the fitted line for all participants. (**b**,**c**) Association between BMD and VAT% stratified by sex and age. Baseline characteristics were adjusted. The subgroup analyses were performed without adjusting for sex and age.

**Figure 5:**
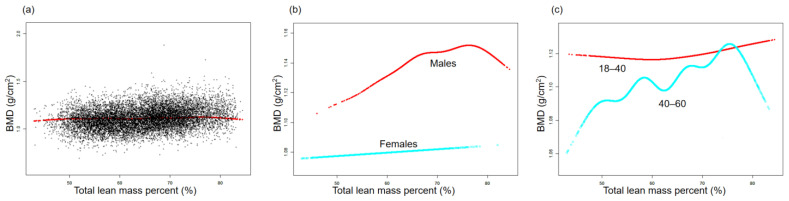
Relationship between BMD and total lean mass percent (%). (**a**) Each black dot on the graph denotes a single sample and the red line represents the fitted line for all participants. (**b**,**c**) Association between BMD and TLM% stratified by sex and age. Baseline characteristics were adjusted. The subgroup analyses were performed without adjusting for sex and age.

The association between BMD and TPF is illustrated by the smooth curve fittings in Figure 1. Fitted relationships between TPF and BMD, stratified by sex and age, revealed a negative correlation in male, female, and middle-aged adult groups, whereas the negative correlation in the young adult group disappeared when TPF exceeded 38.2% (Figure 1b,c). Figure 2 depicts the non-linear relationship between BMD and APF. Upon stratification by sex, BMD in females did not show a significant correlation with APF. In males, BMD and APF exhibited a curvilinear relationship with an inverted U-shape, reaching a peak at 35.6% APF (Figure 2b). In terms of the subgroups stratified by age, the negative association between BMD and APF in the young adult group was consistent with that shown in Table 2. In the middle-aged adult group, an inflection point was observed at 31.7% APF (Figure 2c). The relationships between GPF, VAT%, and BMD were further analyzed using curve-fitting algorithms. The results consistently showed negative correlations among GPF, VAT%, and BMD, which also persisted when the data were stratified by age and sex (Figure 3 and Figure 4). Figure 5 illustrates a positive relationship between TLM% and BMD in females, while in males, there was no significant positive correlation between TLM% and BMD beyond 68.6% of TLM%.

## 4. Discussion

Our study revealed significant negative correlations between BMD and adiposity measures (TPF, APF, GPF, and VAT%) and positive correlations between BMD and TLM% in adults aged 18–60 years by analyzing NHANES data from 1999 to 2018. Upon stratifying the data by age and sex, only APF exhibited no significant correlation with BMD in the female or young adult groups, whereas the other three indices of adiposity maintained statistically significant negative correlations with BMD in each subgroup. Notably, in the young adult group, exceeding a TPF of 38.2% eliminated the negative correlation between BMD and TPF. Furthermore, there was a distinct inverted U-shaped correlation between BMD and APF in males, with an inflection point of 35.6%. Similarly, the APF in the middle-aged adult group displayed an inverted U-shaped relationship, reaching an inflection point of 31.7%. Regarding lean tissue, the positive correlation between TLM% and BMD remained consistent among females. However, in males, once TLM% surpassed 68.6%, the previously observed positive correlation between TLM% and BMD ceased to exist.

Although the association between obesity and BMD has garnered significant interest, its impact remains controversial. Many studies have proposed that obesity may confer protective effects on bone health, whereas lower BMI has been associated with decreased BMD and an elevated risk of osteoporosis [1,7,9]. Salamat et al. conducted an analysis involving 5892 participants, examining their BMI as well as hip and spine BMD. The study findings indicated that obese individuals exhibited higher BMD than those of normal weight [19]. Evans et al. conducted a study focusing on individuals aged 55–75 years and found that obese participants exhibited higher BMD compared to participants with a normal BMI [8]. This suggests that obesity might play a protective role in preventing age-related bone loss. However, a contrasting perspective emerged in research carried out by Sukumar et al., who examined the BMI and BMD of 211 adult women. Their findings revealed that elevated BMI was linked to increased trabecular BMD but decreased cortical BMD [20]. Cohen et al. supported our findings by demonstrating a negative association among trunk fat, bone formation rates, and trabecular bone volume in premenopausal women. Despite adjusting for age and BMI, the negative association between trunk fat and BMD remained significant [21]. Bredella et al. also observed a significant negative correlation between VAT and BMD. However, this study uncovered a positive connection between BMD and lean mass in adult women [22]. In the meantime, Hammoud et al. demonstrated that the lean mass exhibited a positive correlation with BMD in premenopausal women, whereas the impact of BMI on BMD was found to be insignificant in this group [23]. Our study also found the positive influence of lean tissue on BMD, providing additional support to the existing findings in this area.

The varied relationships observed between obesity and BMD in previous studies can be attributed to several factors, including disparities in the measurement methods employed to assess obesity. Body composition measurements obtained through DXA are more precise and informative than those based solely on BMI [5]. Moreover, most evidence supporting the interplay between obesity and BMD is derived from large-scale observational studies, which are inherently incapable of establishing causality and are subject to inherent limitations. Additional factors contributing to these disparities include limited sample sizes, variations in BMD measurement locations, and numerous confounding variables within the study population. Although a comprehensive understanding of the bone–obesity–muscle relationship remains elusive, it is evident that a complex association exists between them [24,25].

Previous studies have postulated that the observed higher BMD in individuals with an elevated BMI might be ascribed to the mechanical loading of weight on bones [26,27]. When subjected to a load, osteocyte apoptosis is delayed, and the stress signal can stimulate osteoblast differentiation while inhibiting osteoclast function [10,28,29]. However, our study yielded contrasting results, indicating a negative correlation between obesity-related factors (TPF, APF, GPF, and VAT%) and BMD.

This discrepancy in the above studies may arise from the fact that the proportion of adipose tissue in relation to the total body weight is typically less than 40% on average [30]. Therefore, the mechanical influence of weight on bones may stem primarily from lean mass rather than from adipose tissue [31,32]. In addition to lean mass, emerging investigations have elucidated a significant association between muscle strength and bone health as stretching muscles also increases the mechanical load on connected bones, leading to a higher BMD [33]. In fact, over recent years, reduced BMD, muscle loss, and obesity are no longer regarded as independent diseases; instead, the concept of ‘osteosarcopenic obesity’ has emerged [32,34]. Many studies have indicated intricate interconnections between lean tissues and bones. On the one hand, the lean tissue releases a diverse array of molecules, including myostatin, osteoglycin, insulin-like growth factor-1, and others, which are believed to exert an influence on bone health; on the other hand, bone-derived factors like osteocalcin will, in turn, affect the synthesis of lean tissue proteins [35,36,37]. Our study substantiated the viewpoint by revealing a substantial positive correlation between TLM% and BMD. Santos et al. also observed that an increase in lean mass directly corresponds to enhanced BMD, whereas sarcopenic obesity may lead to osteoporosis [38]. Despite the close relationship between bone, obesity, and muscle, it is uncommon to encounter cases with simultaneous involvement of all three tissues, possibly owing to varying diagnostic criteria for osteosarcopenic obesity [34,39]. Consequently, most studies have concentrated on exploring the relationship between two of these tissues, with recent extensive discussions revolving around the link between adiposity and BMD [40,41].

Estrogen also has a significant impact on the association between obesity and BMD. Extensive studies have demonstrated that estrogen significantly influences bone metabolism by promoting bone formation and reducing bone resorption [13,14,42]. It is worth noting that adipose tissue can generate aromatase, a synthetic estrogen precursor [42]. Consequently, postmenopausal women who are obese tend to exhibit higher estrogen levels than those with normal weight [14]. However, recent studies have revealed contrasting effects of different adipose tissues on BMD [15,21,43]. VAT has been verified to be linked to increased cortical porosity and decreased bone formation, leading to a decline in BMD [21]. Conversely, subcutaneous adipose tissue appears to positively affect bone tissue [44].

Over the past few decades, obesity has gained recognition as a metabolic abnormality that disrupts the overall metabolism of the body and causes mild inflammation due to various hormones and inflammatory cytokines related to adipose tissue [2,45]. Adipose tissue, lean tissue, and bones are closely interconnected in their metabolic functions [46]. Leptin plays a significant role in the regulation of BMD. Its primary source of secretion is the white adipose tissue, and it is discovered at high concentrations among individuals with obesity [47]. Leptin exerts a dual effect on bone health. In vitro studies demonstrated its ability to hinder osteoclast formation and promote stromal cells to differentiate into osteoblasts [47,48]. Notably, Bao et al. observed a decrease in both BMD and volume in leptin gene knockout mice [49]. However, elevated leptin levels are associated with reduced serotonin production by hypothalamic neurons, thereby impairing bone formation [50]. Overall, leptin exerted a predominantly negative effect on the bone tissue [51,52]. Adiponectin, an adipokine secreted by white adipose tissue, has been reported to enhance bone marrow mesenchymal stem cells to differentiate into osteoblasts through up-regulating CXCL1 and CXCL8 [53]. Interestingly, adiponectin levels tend to be lower in obese individuals [54]. Furthermore, increasing levels of inflammatory cytokines like IL-1, IL-6, and TNF-α are associated with obesity, and these cytokines are secreted by the adipose tissue [55,56]. TNF-α can increase RANKL expression, leading to enhanced bone resorption by osteoclasts [57]. Similarly, IL-6 can induce osteoclast formation and enhance bone resorption [56]. Additionally, factors related to adipose tissue, including resistin, peroxisome proliferator-activated receptor gamma, and peptide YY, are believed to participate in the detrimental influence of obesity on bone health [58,59,60]. Notably, obesity is also related to a higher prevalence of secondary hyperparathyroidism, characterized by increased concentrations of parathyroid hormones, which further contribute to decreased BMD [61].

Furthermore, our study identified sex- and age-related differences in certain fat distribution indices and TLM%; however, the precise reasons behind these differences remain unclear. It is widely acknowledged that the risk of osteoporosis is significantly higher in postmenopausal women when compared to premenopausal women and men [52]. The abrupt decline in estrogen levels among postmenopausal women is considered to be the primary factor contributing to this phenomenon [14]. Additionally, sex differences in android fat, which is recognized as a predictor of metabolic syndrome (MetS), have been observed. Observational research conducted by Bi et al. revealed a notable negative relationship between android fat percentage and the risk of MetS in women, whereas a significant positive relationship was observed in men [15]. This difference in sexes could be attributed to differences in fat distribution. When GPF levels are similar, men tend to exhibit higher APF levels, suggesting a greater tendency for upper body fat accumulation [15]. In our large-scale study, we consistently found a positive correlation between TLM% and BMD among women. However, in men, this positive correlation disappeared when TLM% exceeded 68.6%. Regarding age-related differences, it is plausible that as individuals age, fat storage sites shift from subcutaneous depots to more detrimental ectopic locations, accompanied by the increased inflammatory response of preadipocytes and immune cells [18,62].

Our study had several advantages. First, we used the NHANES to collect extensive data on fat distribution (TPF, APF, GPF, and VAT%), lean tissue (TLM%), and BMD from a representative sample of American adults on a national scale. Our large sample size allowed us to analyze the correlation between obesity and BMD by considering sex and age differences. Additionally, the use of an accurate DXA technique to measure the fat content added credibility to our findings.

However, there are a few limitations in this study. Firstly, the existence of a causal relationship between BMD and obesity could not be determined as this was an observational study, and long-term follow-up research is needed to demonstrate these findings. Furthermore, the sex and age differences we observed, along with the inverted U-shaped curve in the APF-male and APF-40–60 age groups, require further investigation for a better understanding. In different age and gender groups, the relationship between TLM% and BMD exhibited varying patterns. Further investigation is essential to gain a comprehensive understanding of how different locations and types of adipose tissue or lean tissue affect bone health. In the future, as diagnostic criteria for osteosarcopenic obesity become more standardized, we can conduct in-depth investigations into the interplay among osteoporosis, sarcopenia, and obesity, with the optimistic aim of pursuing integrated therapeutic strategies.

In summary, our study highlights the intricate association between BMD and body composition indexes, including fat distribution and total lean mass. However, the precise roles and mechanisms through which obesity or lean tissue influence the development of adult BMD require further investigation. The connection between BMD and obesity is bidirectional and encompasses various factors including mechanical loading, estrogen levels, metabolic factors, and sex and age differences. Advanced research in this area holds promise for enhancing our understanding of this relationship and offers valuable insights into the prevention and management of obesity and osteoporosis.

## 5. Conclusions

This study revealed a negative relationship between BMD and adiposity measures, including TPF, APF, GPF, and VAT%, in individuals aged 18–60 years. Additionally, our study revealed a significant positive correlation between TLM% and BMD in adults. In the young adult subgroup, a TPF exceeding 38.2% abolished the negative correlation between TPF and BMD. In the male and middle-aged adult subgroups, APF and BMD exhibited a distinct inverted U-shaped non-linear relationship. This large-sample study discovered a significant negative association between adiposity measures and BMD, providing valuable perceptions of the intricate connection between adiposity and bone health.

## Figures and Tables

**Table 1:** Baseline characteristics of participants by gender.

Characteristics	Males	Females
Participants (No.)	5847	5768
BMD (g/cm^2^)	1.15 ± 0.10	1.08 ± 0.10
Gynoid percent fat (%)	28.58 ± 5.67	42.18 ± 5.27
Android percent fat (%)	31.23 ± 8.38	38.20 ± 8.48
Total percent fat (%)	27.03 ± 6.12	38.47 ± 6.34
Visceral adipose tissue percent (%)	29.98 ± 8.19	18.33 ± 6.08
Total lean mass percent (%)	69.91 ± 5.69	58.64 ± 5.87
Age (years)	38.07 ± 12.33	38.80 ± 12.16
Income-to-poverty ratio	2.95 ± 1.67	2.88 ± 1.70
BMI (kg/m^2^)	28.36 ± 5.80	28.68 ± 7.28
Waist circumference (cm)	98.66 ± 15.31	94.79 ± 16.60
Arm circumference (cm)	34.34 ± 4.33	31.99 ± 5.28
Total Cholesterol (mmol/L)	4.90 ± 1.06	4.93 ± 1.05
Triglycerides (mmol/L)	1.92 ± 1.84	1.43 ± 1.27
LDL Cholesterol (mmol/L)	2.95 ± 0.88	2.90 ± 0.88
HDL Cholesterol (mmol/L)	1.24 ± 0.35	1.50 ± 0.41
Creatinine (µmol/L)	85.41 ± 29.39	65.50 ± 18.77
Serum uric acid (µmol/L)	357.60 ± 72.43	273.85 ± 64.96
Blood urea nitrogen (mmol/L)	4.88 ± 1.55	4.21 ± 1.43
Race (%)		
Non-Hispanic White	60.29	61.69
Non-Hispanic Black	11.19	11.56
Hispanic	18.73	17.22
Others	9.80	9.53
Diabetes (%)		
Yes	7.20	6.82
No	92.80	93.18
Hypertension (%)		
Yes	22.80	19.58
No	77.20	80.42
Hyperlipidemia (%)		
Yes	26.19	21.87
No	73.81	78.13
Smoking status (%)		
Yes	45.75	34.20
No	54.25	65.80
Vigorous work activity (%)		
Yes	34.70	15.32
No	65.30	84.68

Continuous variables were presented as mean ± SD, and the *p*-value was derived using a weighted linear regression model. Categorical variables were presented as percentages, and the *p*-value was derived through a weighted chi-square test.

**Table 2:** The relationship between BMD and body composition indexes in adults of different genders and ages.

Outcome: BMD	Exposures β (95% CI) *p*-Value				
	TPF (%)	APF (%)	GPF (%)	VAT% (%)	TLM% (%)
	−0.001 (−0.002, −0.001) <0.00001	−0.000 (−0.001, −0.000) 0.00004	−0.002 (−0.002, −0.001) <0.00001	−0.002 (−0.002, −0.002) <0.00001	0.001 (0.000, 0.001) <0.00001
Sex					
Males	−0.002 (−0.002, −0.001) <0.00001	−0.001 (−0.001, −0.001) <0.00001	−0.002 (−0.002, −0.001) <0.00001	−0.002 (−0.002, −0.002) <0.00001	0.001 (0.001, 0.002) <0.00001
Females	−0.001 (−0.001, −0.000) 0.00110	−0.000 (−0.000, 0.000) 0.41294	−0.002 (−0.002, −0.001) <0.00001	−0.002 (−0.002, −0.001) <0.00001	0.000 (−0.000, 0.001) 0.36414
Age					
18–40 years	−0.001 (−0.001, −0.000) 0.00006	−0.000 (−0.000, 0.000) 0.13538	−0.001 (−0.002, −0.001) <0.00001	−0.001 (−0.002, −0.001) <0.00001	0.000 (−0.000, 0.001) 0.05500
40–60 years	−0.002 (−0.003, −0.002) <0.00001	−0.001 (−0.001, −0.001) <0.00001	−0.002 (−0.003, −0.002) <0.00001	−0.003 (−0.003, −0.002) <0.00001	0.001 (0.001, 0.002) <0.00001

TPF, total percent fat; APF, android percent fat; GPF, gynoid percent fat; VAT%, visceral adipose tissue percent; and TLM%, total lean mass percent. Baseline characteristics were adjusted. The subgroup analyses were performed without adjusting for sex and age.

## Data Availability

This study analyzed publicly available datasets. The data can be found at http://www.cdc.gov/nchs/nhanes/ (accessed on 23 May 2023).
